# Activation of TRPV1 and TRPM8 Channels in the Larynx and Associated Laryngopharyngeal Regions Facilitates the Swallowing Reflex

**DOI:** 10.3390/ijms19124113

**Published:** 2018-12-18

**Authors:** Mohammad Zakir Hossain, Hiroshi Ando, Shumpei Unno, Yuji Masuda, Junichi Kitagawa

**Affiliations:** 1 Department of Oral Physiology, School of Dentistry, Matsumoto Dental University, 1780 Gobara Hirooka, Shiojiri, Nagano 399-0781, Japan; shumpei.unno@mdu.ac.jp; 2 Department of Biology, School of Dentistry, Matsumoto Dental University, 1780 Gobara, Hirooka, Shiojiri, Nagano 399-0781, Japan; hiroshi.ando@mdu.ac.jp; 3 Institute for Oral Science, Matsumoto Dental University, 1780 Gobara, Hirooka, Shiojiri, Nagano 399-0781, Japan; yuji.masuda@mdu.ac.jp

**Keywords:** TRPV1, TRPM8, Superior laryngeal nerve, Swallowing reflex, Dysphagia

## Abstract

The larynx and associated laryngopharyngeal regions are innervated by the superior laryngeal nerve (SLN) and are highly reflexogenic. Transient receptor potential (TRP) channels have recently been detected in SLN innervated regions; however, their involvement in the swallowing reflex has not been fully elucidated. Here, we explore the contribution of two TRP channels, TRPV1 and TRPM8, located in SLN-innervated regions to the swallowing reflex. Immunohistochemistry identified TRPV1 and TRPM8 on cell bodies of SLN afferents located in the nodose-petrosal-jugular ganglionic complex. The majority of TRPV1 and TRPM8 immunoreactivity was located on unmyelinated neurons. Topical application of different concentrations of TRPV1 and TRPM8 agonists modulated SLN activity. Application of the agonists evoked a significantly greater number of swallowing reflexes compared with the number evoked by distilled water. The interval between the reflexes evoked by the agonists was shorter than that produced by distilled water. Prior topical application of respective TRPV1 or TRPM8 antagonists significantly reduced the number of agonist-evoked reflexes. The findings suggest that the activation of TRPV1 and TRPM8 channels present in the swallowing-related regions can facilitate the evoking of swallowing reflex. Targeting the TRP channels could be a potential therapeutic strategy for the management of dysphagia.

## 1. Introduction

The pharyngeal and laryngeal regions are important areas for swallowing, respiration, and phonation, and are sources of vital reflexes, such as the swallowing reflex [1,2,3,4,5,6]. These regions are innervated by glossopharyngeal (IX) and vagus (X) nerves [6,7,8,9,10,11]. The pharyngeal region is supplied by a nerve-plexus, named as the pharyngeal plexus, mainly formed by the pharyngeal branches of glossopharyngeal (IX-ph) and vagus (X-ph) nerves [6,7]. The larynx and associated laryngopharyngeal regions are mainly supplied by the superior laryngeal nerve (SLN), a branch of the vagus (X) nerve [8,9,10,11,12]. Sensory branches of the SLN innervate the anterior wall of the laryngopharynx, the laryngeal surface of the epiglottis, the aryepiglottic folds, and the larynx as far as the vocal folds and upper esophagus [2,6,8,9,10,11,12]. The cell bodies of sensory nerves innervating the larynx and pharynx in rats are distributed in the nodose-petrosal-jugular ganglionic complex (NPJc), which consists of nodose (NG), petrosal (PG), and jugular (JG) ganglia [11,12]. The sensory nerves innervating the pharyngeal and laryngeal regions respond to various chemical stimuli, including acids and salts [13,14,15,16,17]. However, the mechanism underlying activation of sensory nerves by chemicals has not been fully elucidated. Transient receptor potential (TRP) channels constitute a large family of ion channels activated by various chemicals and temperature changes, and are involved in various physiological functions [18,19,20]. TRP channels are present in the pharynx, larynx, and NPJc [20,21,22]. Immunohistochemistry showed localization of transient receptor potential vanilloid 1 (TRPV1) and vanilloid 2 (TRPV2) in the pharynx [22]. Transient receptor potential melastatin 8 (TRPM8)-immunoreactive (IR) nerve fibers are present in the posterior portion of the soft palate and in the border region of the naso–oral and laryngeal parts of the pharynx [21]. TRPM8-IR was also observed on the mucosa of the larynx and on the laryngeal side of the epiglottis [21]. TRPV1 channels are also present in the human oropharynx and larynx [23,24]. The presence of TRP channels in these regions raises the possibility of their involvement in the responses of the sensory nerves to various chemicals. 

The SLN, which supplies the larynx and associated laryngopharyngeal regions, plays an important role in the swallowing reflex [1,2,4,5,8]. Mechanical or electrical stimulation of SLN-innervated regions readily evokes the swallowing reflex [4,5,6,25]. The mucosa of these regions is much more highly innervated than the skin or the mucosa of the oral cavity [26]. The mucosa of these regions is richly innervated by free nerve endings [27,28] and many of the sensory nerve fibers of the SLN are unmyelinated [29]. It is highly likely that these unmyelinated nerve fibers are involved in evoking the swallowing reflex. The swallowing reflex is a vital reflex that not only allows the passage of food and drink into the stomach but also prevents their entry into the lungs [30,31]. Dysfunction of the swallowing reflex (oropharyngeal dysphagia) is a major health problem among elderly people and people with neurological disorders (e.g. Parkinson’s and Alzheimer’s diseases, and following a stroke) [32,33]. Therefore, potential strategies that can facilitate the swallowing reflex are important. 

Previous studies observed that the application of capsaicin incorporated in a solution, troche, or food [34,35,36] or adding of natural capsaicinoids in alimentary bolus [37,38] reduced the delay of the swallowing reflex and improved the safety and efficacy of swallowing in human patients with dysphagia. Infusion of a menthol solution in the pharynx (10 mM menthol showed best response) [39] and adding of menthol in alimentary bolus [38] also reduced the delay of the swallowing reflex [39] and the laryngeal vestibule closure time (an indicator of swallowing safety) [38] in human patients with dysphagia. The findings of these studies suggest that TRPV1 and TRPM8 agonists can improve swallowing function. However, as those studies were conducted in human subjects, specific involvement of TRPV1 and TRPM8 channels could not be tested by using pharmacological antagonists of the respective channels. In addition, evaluating the nerve activity that supply the pharynx and laryngeal regions following application of agonists and antagonists, and the amount or pattern of expression of TRPV1 and TRPM8 channels in the NPJc was not possible. Animal model use in the present study allows us to understand the underlying mechanism of capsaicin and menthol evoked improvement of the swallowing function observed in human studies. It also allows us to focus on a particular nerve (SLN) that innervates the larynx and associated laryngopharyngeal regions.

In the present study, we explored whether TRPV1 and TRPM8 are present in the afferent nerves innervating the larynx and associated laryngopharyngeal regions, whether activation of these channels can modulate SLN activity, and whether activation of these channels has any effect on the swallowing reflex. 

## 2. Results

### 2.1. TRPV1 and TRPM8 Expression in the NPJc

**Figure 1:**
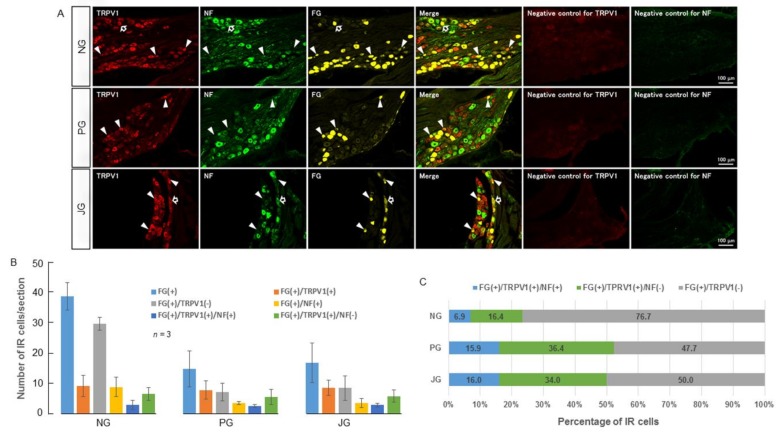
(**A**) Photomicrographs of TRPV1 localization in the NPJc. Black-filled arrows indicate examples of cells positive for FG, TRPV1 and NF-200. White arrowheads indicate examples of cells positive for both FG and TRPV1, but negative for NF-200. (**B**) Number of TRPV1-positive cells in nodose (NG), petrosal (PG), and jugular (JG) ganglia. (**C**) Percentage of TRPV1-positive cells in nodose (NG), petrosal (PG), and jugular (JG) ganglia. FG(+): Cells stained with FG. FG(+)/TRPV1(+): FG-stained cells immune-positive for TRPV1. FG(+)/TRPV1(−): FG-stained cells immune-negative for TRPV1. FG(+)/NF(+): FG-stained cells immune-positive for NF-200. FG(+)/TRPV1(+)/NF(+): FG-stained cells immune-positive for TRPV1 and NF-200. FG(+)/TRPV1(+)/NF(−): FG-stained cells immune-positive for TRPV1 but not NF-200. NPJc: nodose-petrosal-jugular ganglionic complex. FG: fluoro-gold. NF-200: neurofilament-200. IR: immunoreactive. Scale bars represent 100 μm.

**Figure 2:**
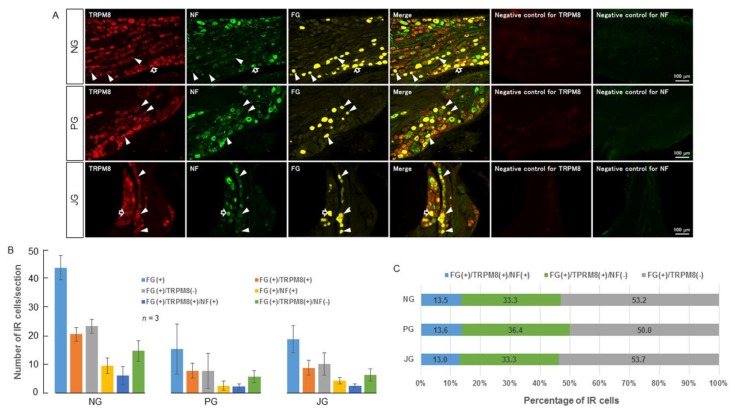
(**A**) Photomicrographs of TRPM8 localization in the NPJc. Black-filled arrows indicate examples of cells positive for FG, TRPM8, and NF-200. White arrowheads indicate examples of cells positive for both FG and TRPM8, but negative for NF-200. (**B**) Number of TRPM8-positive cells in nodose (NG), petrosal (PG), and jugular (JG) ganglia. (**C**) Percentage of TRPM8-positive cells in nodose (NG), petrosal (PG), and jugular (JG) ganglia. FG(+): Cells stained with FG. FG(+)/TRPM8(+): FG-stained cells immuno-positive for TRPM8. FG(+)/TRPM8 (−): FG-stained cells immuno-negative for TRPM8. FG(+)/NF(+): FG-stained cells immuno-positive for NF-200. FG(+)/TRPM8(+)/NF(+): FG-stained cells immuno-positive for TRPM8 and NF-200. FG(+)/TRPM8(+)/NF(−): FG-stained cells immuno-positive for TRPM8 but not NF-200. NPJc: nodose-petrosal-jugular ganglionic complex. FG: fluoro-gold. NF-200: neurofilament-200. IR: immunoreactive. Scale bars represent 100 μm.

TRPV1 and TRPM8 immunoreactivity was observed in the cell bodies present in the nodose, petrosal, and jugular ganglia (Figure 1 and Figure 2). Around 23−52% of fluoro gold-stained neurons expressed TRPV1 (Figure 1C) and around 46−50% of fluoro gold-stained neurons expressed TRPM8 (Figure 2C). Neurons expressing TRPV1 or TRPM8 that did not express NF-200 were more than twice as numerous as neurons that expressed TRPV1 or TRPM8 and NF-200 (Figure 1C and Figure 2C).

### 2.2. SLN Response to Stimulating Solutions

**Figure 3:**
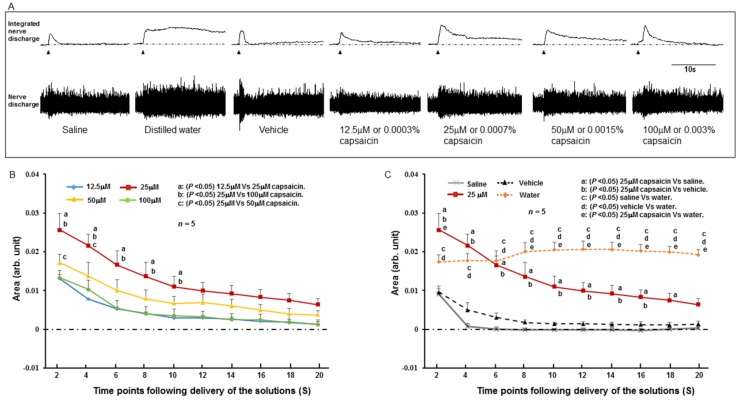
(**A**) The response of the superior laryngeal nerve (SLN) to capsaicin and other stimulating solutions. Arrowheads indicate onset of delivery of the stimulating solutions. (**B**) SLN responses to different concentrations of capsaicin. (**C**) Comparison of SLN responses to capsaicin, saline, vehicle, and distilled water. The y-axis shows the area of the integrated SLN response calculated for every 2 s from the onset of infusion of stimulating solutions. Arb. Unit: arbitrary unit. S: seconds.

**Figure 4:**
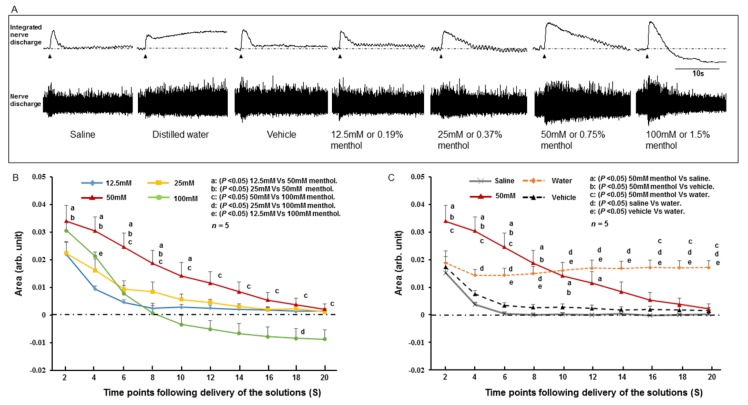
(**A**) The response of the superior laryngeal nerve (SLN) to menthol and other stimulating solutions. Arrowheads indicate onset of delivery of the stimulating solutions. (**B**) SLN responses to different concentrations of menthol. (**C**) Comparison of SLN responses to menthol, saline, vehicle, and distilled water. The y-axis shows the area of the integrated SLN response calculated for every 2 s from the onset of infusion of stimulating solutions. Arb. Unit: arbitrary unit. S: seconds.

The SLN showed spontaneous activity during resting conditions (baseline activity). There was an initial increase of SLN activity upon delivery of the stimulating solutions (Figure 3A and Figure 4A). Following the initial response, the increased SLN activity gradually returned towards baseline levels for all stimulating solutions except for distilled water. For distilled water, the increased SLN activity persisted for a long time and did not returned to the baseline level (Figure 3 and Figure 4). When SLN activities evoked by different concentrations of capsaicin were compared, the highest activity was observed for 25 μM capsaicin (Figure 3A,B). The SLN activity for 25 μM capsaicin was significantly more (*P* < 0.05) than that for 12.5 μM and 100 μM capsaicin until 10 s after delivery of the solution (Figure 3B). The activity was also significantly higher (*P* < 0.05) than that produced by saline or vehicle (Figure 3C) until 18 s after delivery of the solution. The SLN activity for 100 μM capsaicin was similar to that for 12.5 μM capsaicin (Figure 3B). When SLN activity was compared among the different concentrations of menthol, 50 mM menthol showed the highest SLN activity (Figure 4A,B). The SLN activity for 50 mM menthol was significantly higher (*P* < 0.05) compared with that produced by 12.5 mM or 25 mM menthol until 10 s after delivery of the stimulating solution (Figure 4B). The SLN activity for 50 mM menthol was also significantly higher (*P* < 0.05) than that for saline or vehicle (Figure 4C) until 12 s after delivery of the solution. For 100 mM menthol, the SLN activity declined following the initial response and the activity decreased below the baseline level (Figure 4A,B). 

### 2.3. Effect of TRPV1 and TRPM8 Antagonists on the SLN Response

**Figure 5:**
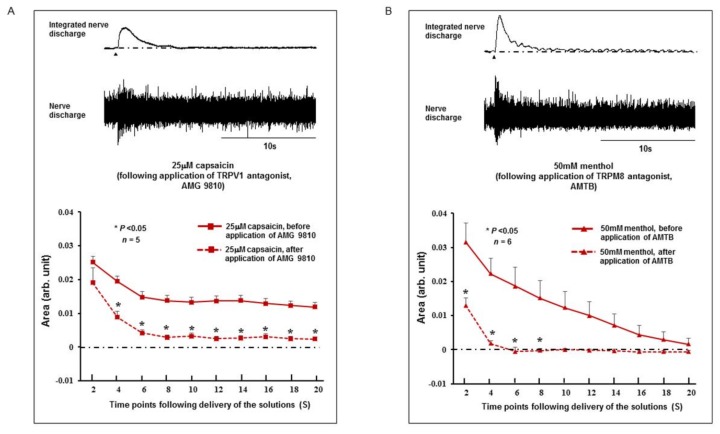
Effect of prior application of TRPV1 and TRPM8 antagonists (AMG 9810 and AMTB as TRPV1 and TRPM8 antagonists, respectively) on respective agonist-evoked superior laryngeal nerve (SLN) responses. (**A**) SLN response following application of TRPV1 antagonist. (**B**) SLN response following application of TRPM8 antagonist. The y-axis shows the area of the integrated SLN response calculated for every 2 s from the onset of infusion of stimulating solutions. Arb. Unit: arbitrary unit. S: seconds.

We investigated the effect of topical application of TRPV1 and TRPM8 antagonists (AMG 9810 and AMTB as TRPV1 and TRPM8 antagonists, respectively) on the SLN response to 25 μM capsaicin and 50 mM menthol, respectively. These concentrations of capsaicin and menthol were chosen because they produced the highest SLN activity (Figure 3B and Figure 4B). Capsaicin or menthol was delivered 10 minutes after application of the TRPV1 or TRPM8 antagonists. As shown in Figure 5A, prior topical application of the TRPV1 antagonist significantly attenuated (*P* < 0.05) capsaicin-evoked SLN activity. Menthol-evoked SLN activity was also significantly attenuated (*P* < 0.05) by prior topical application of the TRPM8 antagonist (Figure 5B).

### 2.4. Swallowing Reflex Evoked by Stimulating Solutions and Effect of TRPV1 and TRPM8 Antagonists on the Reflex

**Figure 6:**
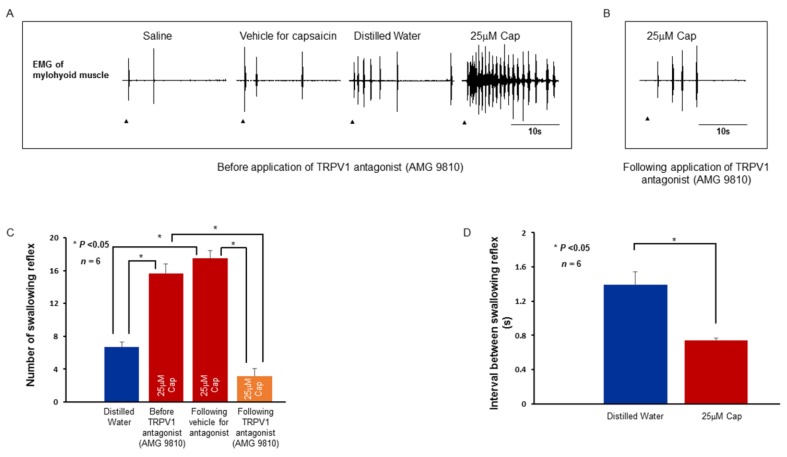
(**A**) Swallowing reflexes evoked by application of capsaicin and other stimulating solutions. Arrowheads indicate onset of delivery of the stimulating solutions. (**B**) Swallowing reflexes evoked by capsaicin following application of TRPV1 antagonist. (**C**) Comparison of the number of swallowing reflexes evoked by distilled water and capsaicin. (**D**) Comparison of the interval between swallowing reflexes evoked by distilled water and capsaicin. Cap: capsaicin.

**Figure 7:**
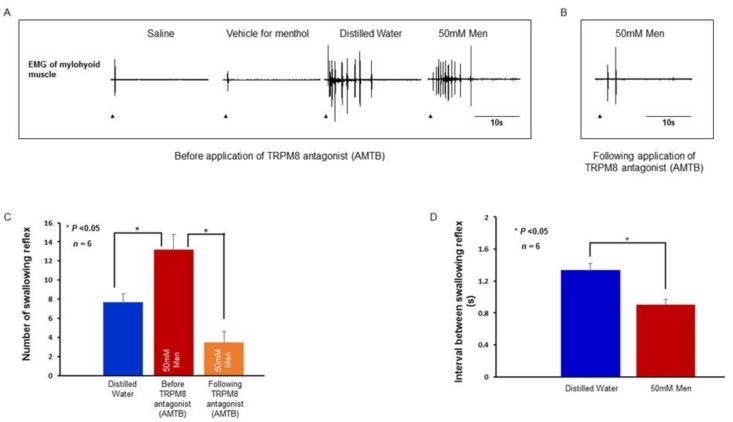
(**A**) Swallowing reflexes evoked by application of menthol and other stimulating solutions. Arrowheads indicate onset of delivery of the stimulating solutions. (**B**) Swallowing reflexes evoked by menthol following application of TRPM8 antagonist. (**C**) Comparison of the number of swallowing reflexes evoked by distilled water and menthol. (**D**) Comparison of the interval between swallowing reflexes evoked by distilled water and menthol. Men: Menthol.

To understand whether activation of TRPV1 and TRPM8 have any effect on the swallowing reflex, we investigated the swallowing reflex evoked by local delivery of the stimulating solutions to the larynx and associated laryngeal regions. Along with distilled water, saline, and vehicle, we used 25 μM capsaicin and 50 mM menthol as stimulating solutions. Delivery of saline or vehicle (saline with small amount of ethanol) evoked 0 to 3 swallowing reflexes (Figure 6 and Figure 7). 

Capsaicin evoked a considerable number of swallowing reflexes and the number of evoked reflexes (15.67 ± 1.17 and 3.17 ± 0.87, before and after TRPV1 antagonist, respectively) was significantly reduced (*P* < 0.001) by prior local application of the TRPV1 antagonist (Figure 6A–C). Application of vehicle for the TRPV1 antagonist did not reduce the number of capsaicin-evoked swallowing reflexes (17.50 ± 0.92) (Figure 6C). The number of swallowing reflexes evoked by capsaicin was significantly more (*P* < 0.001) than that evoked by distilled water (6.67 ± 0.67) (Figure 6C). The interval between the evoked swallowing reflexes was significantly shorter (*P* = 0.002) for capsaicin (0.73 ± 0.03 s) compared with that for distilled water (1.39 ± 0.15 s) (Figure 6D). Menthol-evoked swallowing reflexes (13.17 ± 1.60) were also significantly more (*P* = 0.014) numerous than those evoked by distilled water (7.67 ± 0.92) and prior topical application of TRPM8 antagonist significantly attenuated (*P* < 0.001) the menthol-evoked swallowing reflexes (3.50 ± 1.12) (Figure 7A–C). The interval between the evoked swallowing reflexes was significantly shorter (*P* = 0.002) for menthol (0.90 ± 0.06 s) compared with that for distilled water (1.33 ± 0.08 s) (Figure 7D). 

### 2.5. Effect of SLN Transection on the Evoked Swallowing Reflex

**Figure 8:**
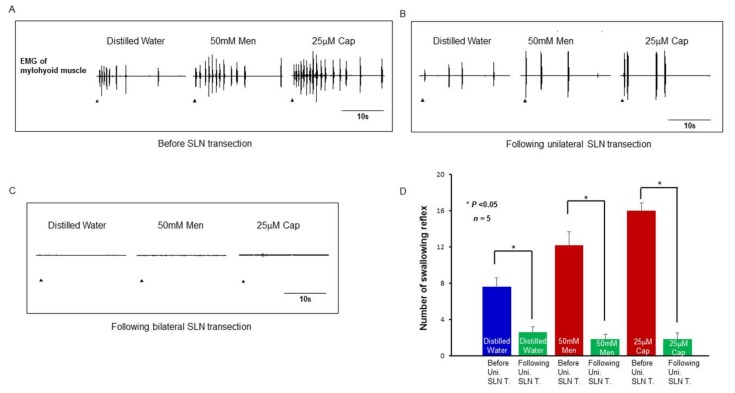
Swallowing reflexes evoked by application of distilled water, capsaicin, and menthol before SLN transection (**A**), following unilateral (**B**) and following bilateral SLN transection (**C**). Arrowheads indicate onset of delivery of the stimulating solutions. (**D**) Comparison of the number of swallowing reflexes before and after unilateral SLN transection evoked by distilled water, menthol, and capsaicin. Men: Menthol, Cap: Capsaicin, Uni. SLN T.: Unilateral transection of the superior laryngeal nerve.

To confirm that the swallowing reflexes evoked by the delivery of stimulating solutions to the larynx and associated laryngopharyngeal region were controlled by the SLN under the experimental setup of this study, we investigated the effect of SLN transection on the number of evoked swallowing reflexes. Unilateral SLN transection significantly reduced (*P* = 0.009, before vs. after unilateral SLN transection for water; *P* < 0.001, before vs. after unilateral SLN transection for capsaicin and menthol) the number of evoked swallowing reflexes, while bilateral SLN transection prevented the swallowing reflex altogether (Figure 8A–D). 

## 3. Discussion

In the present study, we investigated the expression pattern of TRPV1 and TRPM8 in the NPJc, the SLN activity following activation of these channels, and the role of these channels in mediating sensory information through the SLN and their contribution to the swallowing reflex. TRPV1 and TRPM8 were expressed in the NPJc, where the cell bodies of afferent neurons from the SLN are located. The majority of neurons in the NPJc that express TRPV1 and TRPM8 were unmyelinated neurons and a minority were myelinated. Topical application of TRPV1 and TRPM8 agonists (capsaicin and menthol, respectively) to the larynx and associated laryngopharyngeal regions modulated SLN activity and evoked the swallowing reflex. The number of swallowing reflexes evoked by TRPV1 or TRPM8 agonists was greater than the number of water-evoked swallowing reflexes. The SLN activity and the swallowing reflexes evoked by capsaicin and menthol were attenuated by TRPV1 and TRPM8 antagonists, respectively. 

We focused on the SLN, therefore, we transected the other nerves (IX-ph, X-ph and RLN) known to be involved in the swallowing reflex [4,6,7,8]. We created a window in the trachea just below the cricoid cartilage to deliver solutions to the SLN-innervated regions. 

Electrical stimulation of the SLN readily elicits the swallowing reflex, indicating an important role of this nerve in the swallowing reflex [5,25,40,41]. The SLN mainly innervates the larynx and associated laryngopharyngeal regions [8,9,10,11,12] and responds to water and various chemicals applied to those regions [13,14,15,16,17]. The molecular basis of this response is not fully understood. Recently, TRP channels were detected in the larynx, its associated regions [20,21,22,23,24], and the NPJc [20], raising the possibility that these channels are involved in the transduction of chemical stimuli. In the present study, we observed that topical application of capsaicin (a TRPV1 activator) and menthol (a TRPM8 activator) to the larynx and associated laryngopharyngeal regions modulated SLN activity. Capsaicin at a dose of 25 μM and menthol at a dose of 50 mM produced higher SLN responses compared with responses to saline or vehicle. The SLN responses to capsaicin and menthol were suppressed by prior topical application of TRPV1 and TRPM8 antagonists, respectively, indicating involvement of these channels in the responses. A high concentration of menthol (100 mM) caused a brief increase in SLN activity followed by a reduction to below the baseline level. The underlying mechanism of this decrease in SLN activity may result from desensitization of TRPM8 channels and inhibition of other ion channels by the high menthol concentration. In vitro studies show that menthol can interact with other ion channels, including voltage-gated sodium channels [42,43]. A biphasic effect of menthol on neuronal discharge was observed in previous in vivo studies, in which spinal dorsal horn neuronal discharge in response to skin cooling was augmented by application of a low concentration of menthol, while a high concentration of menthol decreased the neuronal discharge and increased the threshold of cold stimuli on the skin [44,45]. Desensitization of TRPM8 by high menthol concentrations may occur via depletion of phosphatidylinositol 4,5-bisphosphate (PIP2) after phospholipase C (PLC) activation [46,47,48] or calcium-mediated activation of calmodulin [49]. In the case of capsaicin, a high concentration (100 μM) did not increase SLN activity but evoked a similar level of activity to that produced by a low concentration of capsaicin. This phenomenon may also be attributed to desensitization of TRPV1 by the high capsaicin concentration [50,51,52]. It has been reported that application of a high concentration or long duration of capsaicin causes high Ca^2+^ influx into the cell and desensitizes the TRPV1 to protect the cell from toxic Ca^2+^ overload. Similar to TRPM8, desensitization may occur by activation of the calcium-binding protein calmodulin that binds with TRPV1 to desensitize it. Depletion of PIP2 and alteration of the dynamic balance between the Ca^2+^-dependent phosphorylation and dephosphorylation of the receptor protein may also contribute to the desensitization [50,51,52].

To understand whether activation of TRPV1 or TRPM8 channels can evoke the swallowing reflex, we stimulated the SLN-innervating regions with 25 μM capsaicin or 50 mM menthol. These two concentrations were chosen because they produced the highest SLN activity (Figure 3B and Figure 4B). Capsaicin and menthol evoked many swallowing reflexes while saline or vehicle evoked no or few swallowing reflexes. Distilled water was previously observed to be an effective stimulus to evoke the swallowing reflex [4,13,25,53,54]. In the present study, we diluted capsaicin and menthol with saline (not with distilled water) to prevent any influence of distilled water on the SLN afferents. We observed that the capsaicin and menthol-evoked swallowing reflexes were greater in number than the water-evoked reflexes, indicating TRPV1 and TRPM8 agonists can be good stimuli to evoke the swallowing reflex. In addition, the number of capsaicin or menthol-evoked swallowing reflexes was significantly reduced following topical application of TRPV1 or TRPM8 antagonists, indicating that these channels are involved. TRPV1 and TRPM8 immunoreactivities in the afferent nerves and epithelial cells of the larynx and associated laryngopharyngeal regions have been reported [20,21,22,23]. 

In our study, we observed that TRPV1 and TRPM8 are expressed in a considerable percentage of afferent neurons from the SLN-innervating regions. They were largely (around two-thirds) expressed in non-NF200-IR neurons, indicating their presence majorly in unmyelinated neurons (C-neurons). Around one-third of TRPV1 and TRPM8 expressing neurons were myelinated neurons. Previous studies also show that generally, TRPV1 and TRPM8 are mainly expressed in unmyelinated neurons and partly in thinly myelinated neurons (Aδ-neurons) [55,56,57,58,59,60]. These observations indicate that facilitation of the swallowing reflex by capsaicin and menthol observed in the present study mostly involved unmyelinated neurons (C-neurons) and partly myelinated neurons (probably Aδ-neurons). The influence of unmyelinated neurons or thinly myelinated neurons (Aδ-neurons) in pharyngeal and laryngeal regions in evoking the swallowing reflex has not been extensively studied. This may be because the swallowing reflex is readily evoked by light mechanical stimulation of the pharyngeal and laryngeal mucosa and by low intensity electrical stimulation of the SLN (mostly activation of Aβ-neurons) [5,6]. In a previous study in human subjects, we observed that low intensity (non-noticeable) electrical stimulation of the pharyngeal regions readily evoked the swallowing reflex and that swallowing reflex latency became shorter as the stimulus frequency increased [3]. These findings indicate that the activation of Aβ-neurons can evoke the swallowing reflex. However, afferent neurons innervating the pharyngeal and laryngeal regions contain a large percentage of unmyelinated neurons (C-neurons) [27,28,29,61]. Facilitation of the swallowing reflex by unmyelinated neurons is of particular interest because utilization of these neurons may be a good strategy for facilitating the swallowing reflex in patients with oropharyngeal dysphagia. Recent studies in patients with oropharyngeal dysphagia reported that addition of a natural capsaicinoid (a TRPV1 agonist, 150 μM) to the alimentary bolus improved the efficacy of swallowing and reduced the prevalence of pharyngeal residues and penetrations of bolus particles into the larynx [37]. It also shortened the laryngeal vestibule closure time and enhanced hyoid motion in oropharyngeal dysphagia patients [37]. In another study in oropharyngeal dysphagia patients, a TRPV1 agonist (capsaicinoid, 150 μM) had a better therapeutic effect on improving swallowing compared with a TRPM8 agonist (menthol, 1 mM or 10 mM) [38]. This study also showed that addition of TRP agonists (capsaicinoid as TRPV1 agonist, 150 μM/menthol as TRPM8 agonist, 1 mM or 10 mM/piperine as TRPA1 and TRPV1 agonists, 150 μM or 1 mM) to the bolus decreased the bolus passing time through the pharynx and reduced the swallowing response time and laryngeal penetrations in dysphagia patients [38]. In addition, in aged people with dysphagia, a TRPV1 or TRPM8 agonist (capsaicin or menthol) applied to the pharynx significantly shortened the latency of the evoked swallowing reflex compared with that for distilled water [39,62]. These studies in humans, along with the findings of the present study, strongly indicate that targeting TRP channels can be a potential new therapeutic avenue to treat oropharyngeal dysphagia.

In the NG, the number of TRPM8-immunoreactive SLN-afferent neurons was significantly higher compared with the number of TRPV1-immunoreactive SLN-afferent neurons (Figure 1 and Figure 2). In the PG and JG, the numbers of TRPV1- and TRPM8-immunoreactive neurons were similar. This observation suggests that NG may contain more TRPM8-immunoreactive neurons that contribute to menthol evoked swallowing reflexes. 

In this study we have separately searched the expression of TRPV1- and TRPM8-immunoreactive neurons in the NPJc. There is a possibility of co-expression of these two channels in the same neurons. It will be interesting to investigate whether these two channels are co-expressed on the same neurons or on different populations of neurons in the NPJc. This information may help to better pharmacological targeting of the channels based on the expression pattern. Future studies should advance our understanding in this context.

We confirmed involvement of the SLN in the evoked swallowing reflex by transection of the SLN. Unilateral transection of the SLN significantly reduced the evoked reflexes while bilateral transection abolished all reflexes, indicating involvement of SLN afferents in the evoked swallowing reflex observed under these experimental conditions. There was an approximately three-fold reduction in the number of swallowing reflexes following unilateral SLN transection, indicating the importance of bilateral SLNs for evoking the swallowing reflex. This observation is supported by our previous study, where bilateral electrical stimulation of the SLN reduced the onset latency and interval time between successive reflexes compared with responses following unilateral electrical stimulation of the SLN [40]. The spatial summation of bilateral afferent inputs may be crucial for evoking the swallowing reflex. Absence of a swallowing reflex following bilateral transection of the SLN confirmed the involvement of SLN afferents in evoking the swallowing reflex under the experimental conditions used in this study.

In the present study, the concentration of the TRPV1 agonist (capsaicin) was 1000 times lower than that of the TRPM8 agonist, and this low TRPV1 agonist dose evoked a larger number of swallowing reflexes, indicating that the TRPV1 agonist may be a better stimulus for evoking the swallowing reflex compared with the TRPM8 agonist. This observation concurs with a study conducted in human patients with dysphagia [38]. The large number of swallowing reflexes evoked by a low dose of TRPV1 agonist may be attributed to a central mechanism working in the swallowing central pattern generator (sCPG). TRPV1 is densely localized in the terminal ends of solitary tract afferents located in the nucleus tractus solitarii (NTS) [60,63] and activation of these afferents causes release of the excitatory neurotransmitter, glutamate, which generates excitatory postsynaptic currents (EPSCs) in the postsynaptic neurons [64,65,66,67,68,69]. In vitro activation of TRPV1-positive solitary tract afferents caused synchronous and long lasting asynchronous release of glutamate in the NTS, while activation of TRPV1-negative afferents caused only synchronous release of glutamate [65,66,67]. In addition, increasing the number of activated TRPV1-positive solitary tract afferents increased the asynchronous release of glutamate [64,66]. Glutamate is an important excitatory neurotransmitter in generating the swallowing reflex [4,5,70]. Considering the above findings, it may be possible that activation of TRPV1-containing afferent fibers by capsaicin caused long lasting release of glutamate in the sCPG, which contributed to enhancement of the swallowing reflex.

We observed that topical application of distilled water in the larynx and associated laryngopharyngeal regions initiated long lasting SLN activity and the activity was significantly higher than the capsaicin and menthol evoked SLN activity at the later time points following application of the solutions (Figure 3 and Figure 4). However, this long lasting activity did not evoke more swallowing reflexes compared with the number of swallowing reflexes evoked by capsaicin or menthol. On the other hand, application of capsaicin (25 μM) or menthol (50 mM) evoked high SLN activity (at early time points following the application) but the activity was not as long lasting as observed for distilled water, however, they produced more swallowing reflexes compared with the number for distilled water. These observations suggest that the evoking of swallowing reflexes was not parallel with the SLN activity. The exact mechanism of this phenomenon is not clear; however, some possibilities can be speculated. The early SLN firing may be more influential to activate the sCPG located in the brainstem for evoking swallowing reflexes. In addition, chemical activation of unmyelinated afferent neurons may release a large amount of excitatory neurotransmitters in the sCPG, responsible for repeated evoking of swallowing reflexes. In this context, in vitro activation of TRPV1-positive solitary tract afferents was observed to cause long lasting asynchronous release of glutamate in the NTS [64,65,66], where the pattern generator for the swallowing reflex is located [4,25]. However, these hypotheses require evaluation in future studies.

## 4. Materials and Methods

### 4.1. Ethical Approval

The protocol of this study was approved by the Animal Care Committee of Matsumoto Dental University (Ref. No. 277, 14 April 2017) and all experiments were carried out in accordance with the guidelines of the committee and the National Institute of Health Guide for the Care and Use of Laboratory Animals. Every effort was made to minimize animal suffering and to reduce the number of animals used.

### 4.2. Animals

All experiments involved male Sprague-Dawley rats weighing 200–300 g. Animals were housed under standard conditions at 22 ± 2 °C, 40 ± 5% relative humidity, and a photoperiod of 12 h light: 12 h dark. Food and water were provided *ad libitum*. 

### 4.3. Surgical Preparation

Rats were anaesthetized with urethane (1.0–1.5 g/kg, administered intraperitoneally) and were fixed in the supine position with adhesive tape. The adequacy of the anaesthesia was checked by noxious pinching of the hind paw to determine if a withdrawal reflex was evoked, and if so, a supplementary dose of urethane was given. A midline incision was made in the ventral surface of the neck. The trachea was isolated from surrounding tissues and a cannula was inserted towards the lungs to maintain respiration. A small area of trachea (ventral portion only) was surgically removed just below the cricoid cartilage to make a window for delivering solutions. This window reduced the pressure produced in the larynx and associated laryngopharyngeal regions during delivery of stimulating solutions.

### 4.4. Transection of IX-ph, X-ph, and Recurrent Laryngeal Nerves

Along with the SLN, the pharyngeal branch of glossopharyngeal (IX-ph) and vagus (X-ph) nerves, lingual branches of glossopharyngeal (IX-li), and the recurrent laryngeal nerves (RLN) of the vagus nerve are also involved in the swallowing reflex [4,6,7,8]. In this study, we focused on the SLN; therefore, pharyngeal branches of glossopharyngeal (IX-ph) and vagus (X-ph) nerves, lingual branches of glossopharyngeal (IX-li) nerves, and recurrent laryngeal nerves (RLN) of the vagus nerve were transected bilaterally prior to recording from the SLN. The IX-ph, IX-li, and X-ph branches were exposed by retraction of the digastric muscle and the horn of the hyoid bone. The recurrent laryngeal nerves (RLN) were exposed from either side of the trachea. Transection of these nerves was performed before exposing the SLN.

### 4.5. Exposing the SLN and Recording of SLN Activity

The sternothyroid muscle was blunt dissected and the SLN was freed from the surrounding tissues. Bilateral SLN were transected near the end, where the SLN joins with the vagus nerve. Bipolar silver wire electrodes (0.1 mm diameter) were then placed on the SLN unilaterally. Liquid silicone was poured over the electrodes to fix the electrode with the nerve. The silicone spread around the electrodes to isolate them from the surrounding tissues. The silicone also prevented the nerve from drying, allowing the nerve to be recorded for a long time [5]. The SLN activity was amplified and integrated with a time constant of 0.3 s. The data were digitized by the Cambridge Electronics Power 1401 data acquisition system (Cambridge Electronic Design Ltd., Cambridge, UK) and stored for later analysis. 

### 4.6. Recording of Swallowing Reflexes

Before recording the swallowing reflex, bilateral SLNs were kept intact and bilateral RLN, IX-ph, X-ph, and IX-li nerves were transected. The swallowing reflex was identified by electromyogram (EMG) activity of the mylohyoid muscle and by characteristic visual observation of laryngeal movement. To record EMG activity during swallowing, bipolar urethane-coated stainless steel fine wire electrodes (Unique Medical Co., Ltd., Tokyo, Japan) were implanted into the mylohyoid muscle and EMG signals were amplified and digitized by the Cambridge Electronics Power 1401 data acquisition system (Cambridge Electronic Design Ltd., Cambridge, UK) and stored for later analysis. 

### 4.7. Stimulating Solutions and Delivery of the Solutions

The stimulating solutions were distilled water (DW), normal saline (0.9% NaCl), menthol (12.5 mM, 25 mM, 50 mM, and 100 mM), capsaicin (12.5 μM, 25 μM, 50 μM, and 100 μM), and vehicle (vehicle for highest concentration of menthol or capsaicin). Menthol and capsaicin (Wako Pure Chemical Industries Ltd. Osaka, Japan) were dissolved in small amounts of 100% ethanol and diluted in normal saline to achieve the desired concentration. The concentration of capsaicin and menthol was determined from a pilot study where we started with different concentrations of capsaicin and menthol and searched for the concentrations that can change the SLN activity. Stimulating solutions were topically delivered using a syringe with a 21 gauge needle with a blunted tip. During delivery of the stimulating solution, the blunted needle tip was placed into the window (created just below the cricoid cartilage) and directed towards the larynx. Fifty microliters of a stimulating solution was delivered in one second. The SLN responses and swallowing reflexes were recorded for 20 seconds after the delivery of the stimulating solutions. The interval between the deliveries of the stimulating solutions was 5 minutes. During this interval time, the delivered solution was aspirated out and saline was delivered and aspirated out several times to wash the region. Pointed pieces of tissue paper were inserted through the window to absorb the remaining saline. All stimulating solutions were applied at room temperature (24–25 °C).

### 4.8. TRPM8 and TRPV1 Antagonists

AMG 9810 (2E)-N-(2,3-Dihydro-1,4-benzodioxin-6-yl)-3-[4-(1,1-dimethylethyl)phenyl]-2-propenamide (Enzo Life Sciences, Inc. NY, USA) and AMTB [N-(3-aminopropyl)-2-{[(3-methylphenyl) methyl]oxy}-N-(2-thienylmethyl)benzamide hydrochloride salt] (Wako Pure Chemical Industries Ltd. Osaka, Japan) were used as TRPV1 and TRPM8 antagonists, respectively. Various in vitro and in vivo studies showed the effeicacy of AMG 9810 and AMTB to block TRPV1 and TRPM8, respectively [71,72,73,74,75,76]. AMG 9810 was dissolved in small amount of DMSO and Tween 80 and diluted in normal saline. Solution containing DMSO, Tween 80, and normal saline was used as vehicle for AMG 9810. AMTB was dissolved in normal saline. Different concentrations of AMG 9810 and AMTB were used to determine the lowest effective concentrations at which they could inhibit and attenuate the nerve response or the number of swallowing reflexes evoked by menthol or capsaicin. The concentration that attenuated the agonist-evoked nerve response or the number of swallowing reflexes to half or less than half, was used as an effective lowest concentration. The effective lowest concentration for AMG 9810 was 125–250 μM. The effective lowest concentration for AMTB was 62.5 μM.

### 4.9. Immunohistochemistry

Fluoro-gold (FG, 4%) was injected bilaterally into the larynx and associated laryngopharyngeal regions and into the SLN under sodium pentobarbital anaesthesia (50 mg/kg, administered intraperitoneally). Before injection of FG, bilateral RLN, IX-ph, X-ph, and IX-li nerves but not the SLN were transected to allow the passage of FG only through the SLN. Three days later, rats were deeply anesthetized and perfused with saline followed by 4% paraformaldehyde. The NPJcs were removed and immersed in the same fixative. The NPJcs were sectioned at a thickness of 16 μm using a cryostat. Sections were incubated with rabbit polyclonal anti-TRPM8 and anti-TRPV1 antibodies (1:5000 for anti-TRPM8 (Cat# ACC-049) and 1:1000 for anti-TRPV1 (Cat #: ACC-030), Alomone, Israel) and a mouse monoclonal anti-neurofilament-200 antibody (1:1000, Cat# N0142, Sigma-Aldrich, USA) overnight, and processed with appropriate secondary antibodies (labelled with Alexa Fluor 594 and 488 (Molecular Probes, USA)). The sections were cover slipped using ProLong Diamond Anti-fade Reagent (Life technologies, USA) and were examined using a BZ-X700 fluorescence microscope (Keyence Corp., Japan). No immunoreactivity of TRPV1 and TRPM8 was observed when primary antibodies for TRPV1 and TRPM8 were omitted during immunohistochemical procedure (Figure 1A and Figure 2A). The same anti-TRPM8, anti-TRPV1, and anti-NF-200 antibodies were used in previous published studies to detect TRPV1-, TRPM8-, and NF-200-immunoreactive neurons in the other ganglia (e.g., in trigeminal ganglia and dorsal root ganglia) in rats [57,77]. Immunoreactive cells were counted from a region of interest using ImageJ software (NIH Image, USA) in a horizontal section where the highest number of TRPV1-IR or TRPM8-IR cells was observed. Three sections from each rat (one with the largest number of labelled cells and the following two serial sections) were used for counting. 

### 4.10. Data Analysis

SLN responses were analysed as the area of the integrated response above the baseline, using Spike2 software (Cambridge Electronic Design Ltd., Cambridge, UK). The area of the integrated SLN response was calculated for every 2 s from the onset of infusion of stimulating solutions for 20 s following the stimulation. The area of stable baseline activity for 2 s (before onset of stimulating solution infusion) was subtracted from the area of every 2 s response following the onset of the stimulation. The number of evoked swallowing reflexes in the 20 s following the application of stimulating solutions was counted. The swallowing reflex was identified by high amplitude electromyogram (EMG) activity of the mylohyoid muscle and by characteristic visual observation of laryngeal movement. Each high amplitude firing in EMG corresponds to 1 swallowing reflex. The average interval between swallowing reflexes was also calculated from the reflexes evoked within the 10 s period following the onset of stimulating solution infusion. The time interval between the starting of high amplitude EMG activity for one swallowing reflex and the starting of high amplitude EMG activity for the subsequent swallowing reflex was used as the interval between the respective swallowing reflexes. When many swallowing reflexes were evoked with relatively very short intervals, the baseline EMG activity of mylohyoid muscle was also increased. In that case, the time point when high amplitude firing of EMG (related to evoked swallowing reflex) exceeded the baseline activity was used for calculating the interval between the swallowing reflexes. 

### 4.11. Statistical Analysis

Comparison of the nerve responses to the different stimulating solutions was carried out using two-way repeated measures ANOVA followed by Tukey’s test. Comparison of the number of evoked swallowing reflexes to the different stimulating solutions and the comparison of nerve responses before and after antagonist treatment was carried out using one-way ANOVA followed by Tukey’s test. The intervals between swallowing reflexes were compared using the *t*-test. Differences were considered significant at *P* < 0.05. All data are presented as the mean ± S.E.M except the data for immunohistochemistry study which are presented as mean ± SD.

## 5. Conclusions

We have demonstrated that TRPV1 and TRPM8 are expressed in afferent neurons from the SLN-innervating regions. Two-third of them are unmyelinated. Local application of agonists of these channels in the SLN-innervating regions modulate the SLN activity and facilitates the evoking of swallowing reflex. The number of agonist evoked swallowing reflexes reduced when the respective channel’s antagonist is applied before application of the agonist. These findings suggest that TRP channels present in the swallowing related regions can be utilized to manage oropharyngeal dysphagia.
